# Secretory Carcinoma of the Parotid Gland Presenting as a Persistent Cystic Parotid Mass: A Case Report

**DOI:** 10.7759/cureus.79230

**Published:** 2025-02-18

**Authors:** Sai Samhitha Avula Balliahgari, Sagar Kansara, Marshall P Stagg

**Affiliations:** 1 Internal Medicine, Baton Rouge General Medical Center, Baton Rouge, USA; 2 Otolaryngology-Head and Neck Surgery, Louisiana State University School of Medicine, Baton Rouge, USA; 3 Oncology, Our Lady of the Lake Regional Medical Center, Baton Rouge, USA

**Keywords:** breast, carcinoma, malignancy, parotid, recurrence, secretory

## Abstract

Secretory carcinoma (SC) of the salivary glands, previously termed mammary analog secretory carcinoma (MASC), is a rare, low-grade malignant tumor of the salivary glands that closely resembles SC of the breast. This case report discusses parotid SC in a 40-year-old female patient who presented with recurrent parotid swelling. The report aims to demonstrate the importance of recognizing this rare tumor, raising awareness, and adding this case to world literature.

## Introduction

Secretory carcinoma (SC) of the salivary glands shares many morphological, immunohistochemical, and molecular features with SC of the breast. It predominantly occurs in the parotid glands but can also occur in the submandibular glands, minor salivary glands, and oral mucosa. It is an indolent tumor that often presents as a slow-growing mass. Overall, it has a good prognosis, just like its counterpart in the breast, with early detection and intervention. First described in 2010 by Skálová et al. [1], the report reviewed 16 cases of salivary gland tumors that resembled acinic cell carcinoma of the salivary glands. This tumor also showed a strong resemblance to the secretory breast carcinoma [2,3]. All but one of the tumors studied during that study tested positive for the presence of a t(12;15)(p13;q25) translocation that results in the generation of an ETS variant transcription factor 6-neurotrophic tyrosine kinase 3 (ETV6-NTRK3) gene fusion which is also a characteristic of SC of the breast. The World Health Organization (WHO) has recognized salivary SC as a separate entity since 2017. Before official recognition, this tumor was often mistaken for acinic cell carcinoma or adenocarcinoma [4]. Although it is a rare tumor, it is not as uncommon as previously believed. We found fewer than 700 cases were reported through a systematic review of SC literature in English by the Journal of Oral and Maxillofacial Surgery in 2021. In this review, 93% of cases presented as a local disease, 0.9% as distant metastasis, and the rest as locoregional metastases [5]. SC has a low recurrence and low mortality rate, and the review study found 48 cases had tumor recurrence, with only 14 deaths [5]. Here, we discuss an atypical presentation of this rare tumor, SC, as a recurrent parotid cyst in a 40-year-old female patient without metastasis to increase awareness that there are variable presentations of this rare tumor. 

## Case presentation

**Figure 1:**
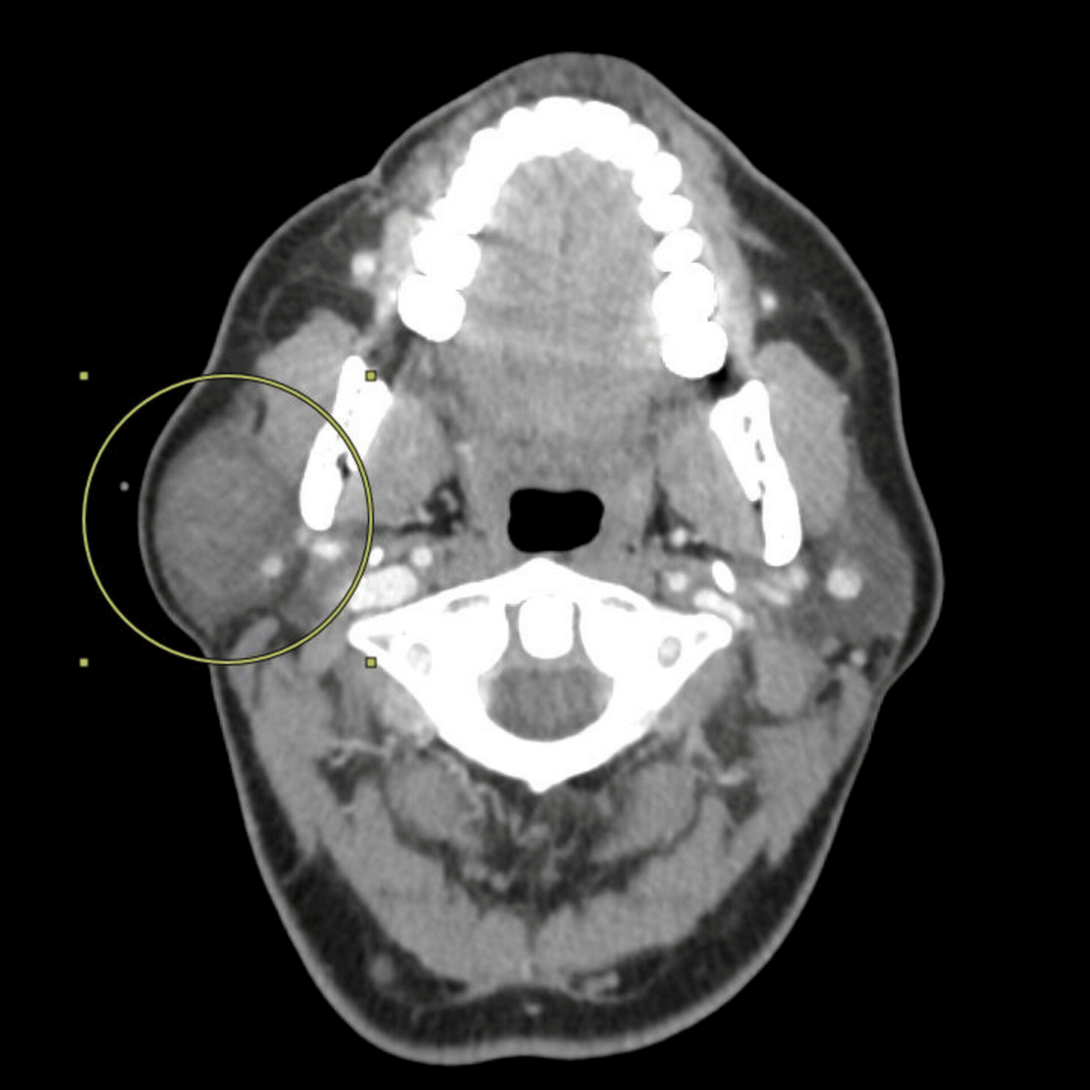
Axial CT of the neck with contrast revealing multiloculated lesion of the right parotid gland with heterogenous cystic and solid appearance. CT: computed tomography

**Figure 2:**
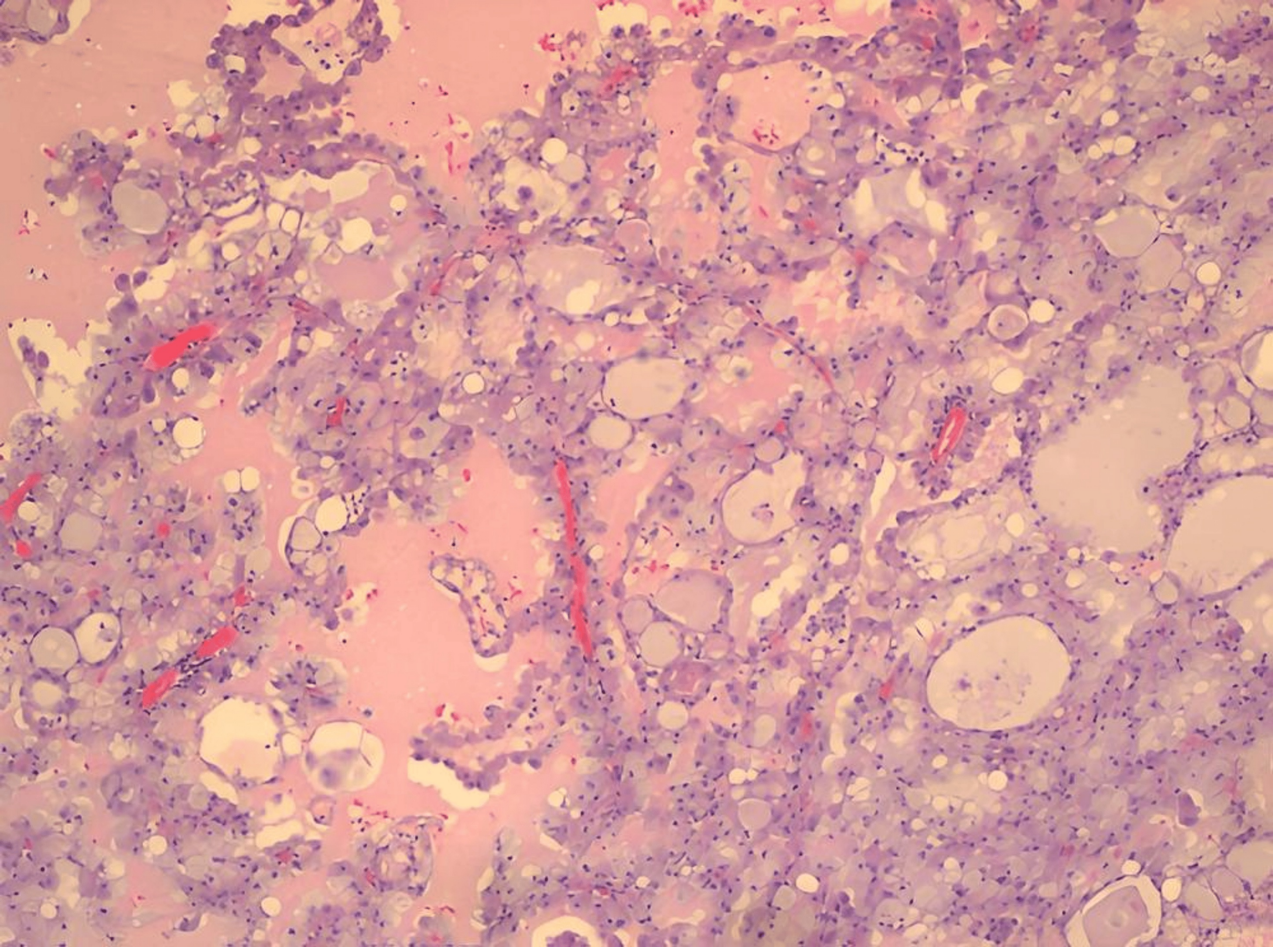
Hematoxylin and eosin staining in 200× magnification revealing uniform tumor cells with centrally located vesicular nuclei and well-developed finely granular eosinophilic or vacuolated cytoplasm with abundant extracellular colloid-like material.

In April 2023, a 40-year-old female patient presented to her primary care physician (PCP) with a slow-growing right parotid mass. She underwent a computed tomography (CT) scan with contrast of the neck in June 2023 that revealed a solid mass with internal vascularity. A biopsy was recommended to rule out malignancy at that time. She presented five months later in November with worsening swelling and underwent drainage of the lesion with a fine needle aspiration that pathologically returned as benign. The swelling recurred four months later, in March 2024; imaging revealed a multiloculated lesion (Figure 1). A repeat fine needle aspiration cytology (FNAC) was performed one month later in April for recurring swelling. This aspiration pathologically was suspicious of malignancy. She underwent parotidectomy in April 2024. The tumor was noted to involve the deep lobe of the parotid gland; careful microdissection of the main trunk of the facial nerve and its branches was required to safely excise the tumor. Pathology confirmed a 2.3 cm (about 0.91 in) secretory adenocarcinoma confined to the parotid gland with clear margins (Figure 2). The tumor was positive for S-100, mammaglobin, low-molecular-weight cytokeratin, cytokeratin AE1/AE3, vimentin, and GATA binding protein 3 (GATA-3) and negative for cytokeratin 5/6, tumor protein 63 (p63), leukocyte common antigen or cluster of differentiation 45 (CD45), paired-box gene 8 (PAX-8), and CD68, which further supported the diagnosis of parotid SC. The extracellular secretions of the tumor cells were also positive for mucicarmine and periodic acid-Schiff (PAS) stain. A positron emission tomography (PET)/CT post-excision showed no significant uptake in the right parotid region or lymph nodes. The patient is currently five months post-excision. Her follow-up will be with clinical examinations and imaging to monitor for recurrence.

## Discussion

SC of the parotid gland, first described in 2010, is a low-grade malignant tumor that strongly resembles SC of the breast. They both express the characteristic diagnostic translocation that results in the fusion gene ETV6-NTRK3 [1]. SC of the salivary glands was later recognized as a separate entity from acinic cell carcinoma in 2017 by WHO, and SC is the official nomenclature for this tumor, previously referred to as mammary analog secretory carcinoma (MASC). This uncommon tumor is likely underappreciated due to its recent discovery and general lack of awareness [6]. SC usually occurs in adults but can present across a wide age range from the second to eighth decades of life with a median presentation age of 45 years, with one case being reported in a patient as young as five years old [7]. It has a slight predilection for males over females [5]. It is a slow-growing and typically painless mass that can occasionally cause pain or facial paralysis due to facial nerve infiltration. It involves the parotid gland about 70% of the time and is less common in other sites, including the submandibular gland, oral cavity, and oropharynx [8-10]. Aggressive cases with extraglandular extension or perineural invasion are uncommon [11]. The Journal of Oral and Maxillofacial Surgery review of 648 cases of salivary gland SC in 2021 reported only 40 cases with locoregional metastasis and six with distant metastasis [5].

SC grossly appears as a discreet, solitary, multilobed mass often encapsulated that can have a cystic component. Histologically, SC can mimic acinic cell carcinoma or adenocarcinoma of the salivary glands [12,13]. It demonstrates various presentations on microscopic examination, including cystic, solid, tubular, or papillary growth patterns [13-16]. The cells appear uniform with centrally located vesicular nuclei and well-developed finely granular eosinophilic or vacuolated cytoplasm. Another notable histologic feature is the presence of zymogen granules and the abundance of extracellular colloid-like material [14-16]. These colloid-like secretions stain positive for PAS and are diastase resistant [14-16]. 

The most common histologic findings in SC include positive staining for cytokeratin 7, S-100, transcription factor SOX-10, vimentin, and mammaglobin and negative staining for transmembrane member 16A (DOG1), nuclear receptor subfamily 4 group A member 3 (NR4A3), P63, P40, and cytokeratin 5/6 [14] according to WHO Head and Neck Tumor Classification 5th Edition [17]. Immunohistochemistry testing of the tumor in the case described above was positive for S-100, mammaglobin, low-molecular-weight cytokeratin, cytokeratin AE1/AE3, vimentin, and GATA-3 and negative for cytokeratin 5/6, p63, CD45, PAX-8, and CD68 consistent with SC. The defining characteristic of SC is the t(12;15)(q13;q25) translocation and fusion of ETV6-NTRK3[18]. This fusion results in a chimeric tyrosine kinase and activates the Ras/mitogen-activated protein (MAP) kinase mitogenic and phosphatidyl inositol-3-kinase-AKT pathways. This ETV6-NTRK3 fusion in SC of the salivary glands is characteristic but not the only fusion described in SC, as other fusions like ETV6-mesenchymal-epithelial transcription factor receptor gene (MET) or ETV6-rearranged during transfection (RET) can occur [19]. 

The numerous similarities between the SC of the breast and salivary glands evoke a question: Is it the same tumor but in a different location, or are they entirely different entities that coincidentally share a few characteristics? This question prompted the WHO to further characterize the similarities between the tumors. The current belief is that they are one entity that shares a similar molecular pathogenesis. With the characteristic translocation, this tumor can present in other locations such as the thyroid and skin. Recognizing this helps to prognosticate and treat these tumors regardless of the location of the primary tumor. The key to early appropriate treatment is the early detection of the t(12;15)(q13;q25) translocation and fusion of ETV6-NTRK3. Treatment with small molecule inhibitors that target NTRK3 and other NTRK genes has already shown a good response in SC of the breast and is a promising therapy for SC in other sites [20]. 

A case discussed by Drilon et al. [20] reported a dramatic tumor response using entrectinib, a pan-Trk inhibitor with in vitro activity against NTRK 1/2/3. However, that patient later acquired a novel mutation in the NTRK3 gene, which caused resistance. The acquired mutation interfered with drug binding, resulting in decreased sensitivity to the drug inhibition of NTRK. A total tumor excision with routine monitoring is the accepted and recommended treatment of choice for SC currently.

## Conclusions

SC usually has a favorable course and a good prognosis. The treatment is similar to other low-grade tumors of the salivary glands. There is no specific chemotherapy or immunotherapy recommended for this tumor, and complete excision of the mass is usually the treatment of choice. There have been a few case reports that discuss the aggressive presentation of SC with metastatic disease, with few deaths reported. However, there is limited information on the long-term prognosis of this malignancy and its metastatic potential. 

This case report attempts to gather and discuss all the relevant information regarding the typical clinical presentation and raise awareness that there are atypical presentations of this rare tumor. We also discuss the management of this uncommon malignancy. The relatively recent recognition of this tumor and the findings in retrospective pathology reviews show that it is not as rare a malignancy as previously believed. Case reports like ours are essential for better understanding the disease course and helping in the prognostication of the disease, the understanding of which is still in its infancy. 
